# Risk of meticillin resistant *Staphylococcus aureus* and *Clostridium difficile* in patients with a documented penicillin allergy: population based matched cohort study

**DOI:** 10.1136/bmj.k2400

**Published:** 2018-06-27

**Authors:** Kimberly G Blumenthal, Na Lu, Yuqing Zhang, Yu Li, Rochelle P Walensky, Hyon K Choi

**Affiliations:** 1 Division of Rheumatology, Allergy, and Immunology, Department of Medicine, Massachusetts General Hospital, Boston, MA 20114, USA; 2 Medical Practice Evaluation Center, Massachusetts General Hospital, Boston, MA, USA; 3 Harvard Medical School, Boston, MA, USA; 4 Division of Infectious Diseases, Department of Medicine, Massachusetts General Hospital, Boston, MA, USA

## Abstract

**Objective:**

To evaluate the relation between penicillin allergy and development of meticillin resistant *Staphylococcus aureus* (MRSA) and *C difficile*.

**Design:**

Population based matched cohort study.

**Setting:**

United Kingdom general practice (1995-2015).

**Participants:**

301 399 adults without previous MRSA or *C difficile* enrolled in the Health Improvement Network database: 64 141 had a penicillin allergy and 237 258 comparators matched on age, sex, and study entry time.

**Main outcome measures:**

The primary outcome was risk of incident MRSA and *C difficile*. Secondary outcomes were use of β lactam antibiotics and β lactam alternative antibiotics.

**Results:**

Among 64 141 adults with penicillin allergy and 237 258 matched comparators, 1365 developed MRSA (442 participants with penicillin allergy and 923 comparators) and 1688 developed *C difficile* (442 participants with penicillin allergy and 1246 comparators) during a mean 6.0 years of follow-up. Among patients with penicillin allergy the adjusted hazard ratio for MRSA was 1.69 (95% confidence interval 1.51 to 1.90) and for *C difficile* was 1.26 (1.12 to 1.40). The adjusted incidence rate ratios for antibiotic use among patients with penicillin allergy were 4.15 (95% confidence interval 4.12 to 4.17) for macrolides, 3.89 (3.66 to 4.12) for clindamycin, and 2.10 (2.08 to 2.13) for fluoroquinolones. Increased use of β lactam alternative antibiotics accounted for 55% of the increased risk of MRSA and 35% of the increased risk of* C difficile*.

**Conclusions:**

Documented penicillin allergy was associated with an increased risk of MRSA and *C difficile* that was mediated by the increased use of β lactam alternative antibiotics. Systematically addressing penicillin allergies may be an important public health strategy to reduce the incidence of MRSA and *C difficile* among patients with a penicillin allergy label.

## Introduction

One third of patients report a drug allergy (ie, adverse or allergic reaction),^1^ the most commonly implicated drug being penicillin and documented in 5-16% of patients.^1^ ^2^ ^3^ ^4^ ^5^ Being labelled with a penicillin allergy affects future prescribing for infections in both outpatients and inpatients, with prescribed antibiotics often more broad spectrum and toxic.^2^ ^6^ ^7^ ^8^ Unnecessary use of broad spectrum antibiotics leads to the development of drug resistant bacteria, including meticillin resistant *Staphylococcus aureus* (MRSA), and healthcare associated infections such as *Clostridium difficile* related colitis.^9^ ^10^ ^11^ ^12^ ^13^

Most patients with a documented penicillin allergy are not allergic—that is, there is no immediate hypersensitivity.^14^ ^15^ After evaluation by an allergist, about 95% of patients with reported penicillin allergies were found to be penicillin tolerant.^14^ The discrepancy between labelled and confirmed penicillin allergy stems from misdiagnosis (eg, a viral exanthem is misinterpreted as an allergy), misassumptions (eg, an intolerance, such as a headache, is listed as an allergy), and remote timing of the allergy evaluation, since 80% of patients with immediate hypersensitivity to penicillin are no longer allergic after 10 years.^16^ Most patients with a penicillin allergy label therefore unnecessarily avoid penicillins, and often other related β lactam antibiotics, such as cephalosporins.^6^ ^7^

To evaluate the public health consequences of having a penicillin allergy label, we conducted a population based matched cohort study and examined the relation between a newly recorded penicillin allergy and the risk of incident MRSA and *C difficile*.

## Methods

### Data source

We used data from the Health Improvement Network (THIN), an electronic medical record database of 11.1 million patients registered with general practices in the United Kingdom. Because the National Health Service requires people to register with a general practice regardless of health status, THIN is a population based cohort representative of the UK general population.^17^ During consultations with patients in primary care, general practitioners (GPs) enter clinical data, including height, weight, smoking status, diagnoses, and prescription drugs. Patient diagnoses are recorded using READ codes, the UK’s standard clinical terminology system.^18^ Drug allergies are linked to a drug prescription, or recorded as a diagnosis (eg, personal history of penicillin allergy). The GP enters details of the drug allergy, including reaction type, severity of reaction, and certainty of diagnosis. All GPs are trained in data entry, with the quality of their data periodically reviewed. Previous studies using THIN have confirmed the validity of both prescriptions and diagnoses.^19^ ^20^

### Study design

We performed a matched cohort study among participants aged more than 18 years, who were enrolled in the THIN database between 1995 and 2015. Eligible participants had no history of MRSA or *C difficile* diagnoses before study entry and were required to have at least one year of enrolment with a general practice before entering the study to allow for assessment of exposure and covariates. We identified adults with their first recorded penicillin allergy and selected up to five penicillin users without a penicillin allergy matched on age (one year either way), sex, and study entry time (within one year either way). Such comparators were chosen to further ensure the comparability of indications for penicillin use (eg, infection tendency) and associated features. The index date for cases was the date of first entry of an allergy diagnosis in the THIN database; the matched index date for comparators was within one year of a penicillin prescription.

### Assessment of exposure and outcomes

The exposure of interest was a documented penicillin allergy, defined as an allergy to a penicillin antibiotic linked to a penicillin prescription, or one or more relevant READ diagnosis codes for a penicillin allergy or adverse effect (see supplemental table 1).

The primary outcomes were incident cases of doctor diagnosed MRSA and *C difficile* during the follow-up period. We identified MRSA and *C difficile* by the presence of one or more relevant READ diagnosis codes.^21^ ^22^ ^23^ ^24^ For MRSA, codes indicated MRSA infection, carriage, eradication, or decontamination whereas for *C difficile*, codes indicated *C difficile* infection or detection of antigen or toxin (see supplementary table 1).

We also assessed antibiotic utilization during the follow-up period, derived from the prescription record. We grouped all antibiotics prescribed into classes: penicillins, first generation cephalosporins, macrolides, clindamycin, fluoroquinolones, tetracyclines, and sulfonamides. Given that vancomycin, aminoglycosides, and linezolid are commonly administered parenterally and therefore seldom administered to outpatients by GPs, we assessed these antibiotics separately.

### Assessment of covariates

We identified demographic and lifestyle factors before the index date, such as age, sex, body mass index, socioeconomic status, smoking status, and alcohol use. READ diagnosis codes at the index date were used to ascertain relevant comorbidities (diabetes, renal disease, hemodialysis, malignancy, liver disease, and infection with human immunodeficiency virus (HIV)) and to calculate the adapted Charlson comorbidity index^25^ at baseline. Using the prescription records, we identified the number of antibiotics prescribed in the year before the index date and whether proton pump inhibitors or systemic corticosteroids were used at baseline. Concomitant allergies to cephalosporin antibiotics and other antibiotics were linked to prescriptions or identified using READ diagnosis codes. We determined participants who were residents of nursing homes at baseline. Finally, we calculated the number of visits to a GP and hospital admissions during the year before the index date.

### Statistical analysis

We compared baseline characteristics between participants with penicillin allergy and their comparators. Follow-up time for each participant was calculated from the index date to the date of one of several events: the study endpoints (MRSA or *C difficile*), death, or end of the study (31 December 2015), whichever occurred first.

We identified incident MRSA cases and number of person years of follow-up for each cohort separately. We calculated the hazard ratios for the relation of penicillin allergy status to the risk of MRSA using Cox proportional hazard models. In the multivariable Cox model we adjusted for age, sex, body mass index, socioeconomic status, smoking status, alcohol use, Charlson comorbidity index, hemodialysis, number of antibiotic prescriptions, proton pump inhibitor use, corticosteroid use, other antibiotic allergies, resident of nursing home, visits to a GP, and admissions to hospital. We repeated the same analyses for the risk of *C difficile*. We also calculated the absolute risk difference.

In both the penicillin allergy cohort and the comparison cohort we determined the rates of subsequent antibiotic utilization by class. We used Poisson regression models to estimate the incidence rate ratio for the relation of penicillin allergy status to the rates of subsequent antibiotic use, while adjusting for the same covariates.

We performed mediation analyses to examine the extent to which the effect of penicillin allergy status on the risk of MRSA or *C difficile* was through its effect on utilization of β lactam alternative antibiotics.^26^ Specifically, we grouped utilization into five categories based on previous studies that evaluated the impact of various antibiotics on the risk of MRSA and *C difficile* 9 10 11 12 13: fluoroquinolones, clindamycin, macrolides, vancomycin, aminoglycosides, and linezolid (all β lactam alternative antibiotics considered in this study); fluoroquinolones, clindamycin, and macrolides; fluoroquinolones and macrolides; fluoroquinolones and clindamycin; and fluoroquinolones alone. Using marginal structural models we then estimated the natural direct effect (ie, the effect of penicillin allergy status on the risk of MRSA or *C difficile* not through a specific group of antibiotics) and the natural indirect effect (ie, the effect of penicillin allergy status on the risk of MRSA or *C difficile* through a specific group of antibiotics), while adjusting for the same confounding variables,^26^ and reported the adjusted risk ratio and percentage mediated.

For all analyses we imputed unknown values for covariates (ie, missing body mass index, alcohol use, and smoking status) using a sequential regression method based on a set of covariates as predictors. To minimize random error, we imputed five datasets and then combined estimates from these datasets by calculating effect estimates from each imputed dataset and then averaging estimates and their confidence intervals using Rubin’s rules.^27^ All analyses were performed using SAS, version 9.2 (SAS Institute, Cary, NC).

### Patient and public involvement

No patients were involved in setting the research question or the outcome measures, nor were they involved in developing plans for implementation of the study. No patients were asked to advise on interpretation or writing up of results. There are no plans to disseminate the results of the research to study participants or the relevant patient community. Individual patient consent was not sought given the use of anonymized data.

## Results

### Cohort identification and characteristics

We identified 64 141 patients with a documented penicillin allergy and 237 258 matched comparators (table 1). Patients with penicillin allergy were identified through allergies linked to prescriptions for penicillin antibiotics (63 245/64 141, 98.6%). Documented penicillin allergies consisted of allergies (74.4%), intolerances (14.5%), and adverse effects (11.1%). Most allergies were considered of moderate severity (86.0%) with likely certainty (73.6%).

**Table 1:** Reactions in patients with penicillin allergy (n=64 141)

Reaction characteristics	No (%)
Type of reaction:	
Allergy	47 698 (74.4)
Intolerance	9300 (14.5)
Adverse effect	7143 (11.1)
Severity:	
Minimal	117 (0.2)
Mild	5193 (8.2)
Moderate	54 372 (86.0)
Severe	3054 (4.8)
Very severe	320 (0.5)
Fatal or life-threatening*	146 (0.2)
Certainty:	
Tentative	176 (0.3)
Unlikely	294 (0.5)
Possible	6812 (10.8)
Likely	46 545 (73.6)
Certain	9141 (14.5)
Absolute	236 (0.4)

* Mean follow-up time of 6.9 years.

Patients with penicillin allergy were similar to their comparators for age, sex, body mass index, socioeconomic status, smoking status, and alcohol use (table 2). They were also similar for diabetes, renal disease, hemodialysis, malignancy, liver disease, HIV, Charlson comorbidity index, previous antibiotic prescriptions, use of proton pump inhibitors and systemic corticosteroids, nursing home residency, visits to a GP, and hospital admissions. Other antibiotic allergies were more common in patients with a penicillin allergy.

**Table 2:** Cohort characteristics according to penicillin allergy status. Values are numbers (percentages) unless stated otherwise

Characteristics	Penicillin allergy (n=64 141)	No penicillin allergy (n=237 258)
Median (interquartile range) age (years)	56.7 (38.4-71.5)	57.1 (38.6-71.7)
Women	43 288 (67.5)	164 773 (69.4)
Body mass index:		
<18.5	1473 (2.3)	4943 (2.1)
18.5-24.9	21 798 (34.0)	82 251 (34.7)
25.0-29.9	17 904 (27.9)	66 578 (28.1)
>30.0	12 455 (19.4)	43 666 (18.4)
Unknown	10 511 (16.4)	39 820 (16.8)
Socioeconomic deprivation index score*:		
1	16 033 (25.3)	58 908 (25.2)
2	13 619 (21.5)	50 317 (21.5)
3	12 863 (20.3)	47 091 (20.2)
4	11 135 (17.6)	40 662 (17.4)
5	7276 (11.5)	27 366 (11.7)
Smoking status:		
None	33 212 (51.8)	124 809 (52.6)
Former	10 107 (15.8)	36 315 (15.3)
Current	17 266 (26.9)	60 930 (25.7)
Unknown	3556 (5.5)	15 204 (6.4)
Alcohol use:		
None	12 245 (19.1)	43 264 (18.2)
Past	859 (1.3)	2575 (1.1)
Current	39 444 (61.5)	146 770 (61.9)
Unknown	11 593 (18.1)	44 649 (18.8)
Comorbidities:		
Diabetes	5453 (8.5)	19 221 (8.1)
Renal disease	4142 (6.5)	14 151 (6.0)
Hemodialysis	67 (0.1)	197 (0.1)
Malignancy	3548 (5.5)	12 217 (5.1)
Liver disease	1096 (1.7)	3378 (1.4)
Human immunodeficiency virus	27 (<0.1)	95 (<0.1)
Median (interquartile range) Charlson comorbidity index	0 (0-1)	0 (0-1)
Medications:		
Median (interquartile range) No of annual antibiotic prescriptions	3 (1-5)	2 (1-4)
Proton pump inhibitor	16 016 (25.0)	54 764 (23.1)
Systemic corticosteroid	21 294 (33.2)	77 312 (32.6)
Other drug allergies:		
Cephalosporins	778 (1.2)	1216 (0.5)
Other antibiotic	5694 (8.9)	10 785 (4.5)
Nursing home resident	71 (0.1)	363 (0.2)
Median (interquartile range) visits to general practitioner	4 (2-7)	4 (2-7)
Median (interquartile range) No of admissions to hospital	0 (0-1)	0 (0-0)

* Townsend deprivation index, grouped into fifths from 1 (least deprived) to 5 (most deprived).

### Penicillin allergy and risk of MRSA and *C difficile*

During the mean follow-up time of 6.0 years for patients with penicillin allergy and 6.1 years for comparator patients, 442 patients with penicillin allergy and 923 comparator patients developed MRSA, and 442 patients with penicillin allergy and 1246 comparator patients developed *C difficile* (table 3 and supplemental table 2).

**Table 3:** Impact of listed penicillin allergy on risk of meticillin resistant *Staphylococcus aureus* (MRSA)and *Clostridium difficile*

Outcomes	Penicillin allergy	No penicillin allergy
MRSA:		
No of patients	64 141	237 258
No of MRSA cases	442	923
Person years	383 199	1 446 753
Hazard ratio (95% CI)*	1.84 (1.64 to 2.06)	1.0 (reference)
Multivariable adjusted hazard ratio (95% CI)†	1.69 (1.51 to 1.90)	1.0 (reference)
*C difficile*:		
No of patients	64 141	237 258
No of *C difficile* cases	442	1246
Person years	383 469	1 446 658
Hazard ratio (95% CI)*	1.37 (1.23 to 1.53)	1.0 (reference)
Multivariable adjusted hazard ratio (95% CI)†	1.26 (1.12 to 1.40)	1.0 (reference)

* Matched on age, sex, and study entry time.

† Matched on age, sex, study entry time and adjusted for age, sex, body mass index, socioeconomic status, smoking status, alcohol status, Charlson comorbidity index, hemodialysis, antibiotic prescriptions, proton pump inhibitor use, corticosteroid use, other antibiotic allergies, nursing home resident, visits to general practitioner, and admissions to hospital.

The age, sex, and study entry time matched hazard ratios for patients with penicillin allergy were 1.84 (95% confidence interval 1.64 to 2.06) for MRSA and 1.37 (1.23 to 1.53) for *C difficile*. The matched and multivariable adjusted hazard ratios for patients with penicillin allergy were 1.69 (1.51 to 1.90) for MRSA and 1.26 (1.12 to 1.40) for *C difficile*, respectively. The corresponding adjusted risk differences were 49/100 000 person years for MRSA and 27/100 000 person years for *C difficile*.

### Penicillin allergy and subsequent antibiotic utilization

Patients with penicillin allergy were less often prescribed penicillin than their comparators (adjusted incidence rate ratio 0.30, 95% confidence interval 0.30 to 0.31), but had increased use of macrolide antibiotics (4.15, 4.12 to 4.17), clindamycin (3.89, 3.66 to 4.12]), fluoroquinolones (2.10, 2.08 to 2.13), tetracyclines (1.75, 1.73 to 1.76), and sulfonamide antibiotics (1.26, 1.25 to 1.27; table 4). Though vancomycin, aminoglycosides, and linezolid were overall infrequently prescribed, they were more often prescribed to patients with penicillin allergy than to their comparators (supplemental table 3).

**Table 4:** Impact of listed penicillin allergy on antibiotic use

Antibiotics	Antibiotic use rate (events/1000 person years)			Incidence rate ratio* (penicillin allergy *v* no penicillin allergy)			
	Penicillin allergy (n=64 141)	No penicillin allergy (n=237 258)		Matched†		Multivariable adjusted‡	
β lactams:							
Penicillins	248.23	751.12		0.32 (0.32 to 0.33)		0.30 (0.30 to 0.31)	
Cephalosporins, 1st generation	165.67	81.16		2.02 (2.00 to 2.04)		1.82 (1.81 to 1.84)	
β lactam alternatives:							
Macrolides	596.32	134.93		4.33 (4.30 to 4.36)		4.15 (4.12 to 4.17)	
Clindamycin	6.35	1.45		4.28 (4.03 to 4.53)		3.89 (3.66 to 4.12)	
Fluoroquinolones	118.44	49.55		2.34 (2.31 to 2.37)		2.10 (2.08 to 2.13)	
Tetracyclines	228.20	119.59		1.86 (1.85 to 1.88)		1.75 (1.73 to 1.76)	
Sulfonamides	193.10	146.66		1.31 (1.29 to 1.32)		1.26 (1.25 to 1.27)	

* All P<0.001.

† Matched on age, sex, and study entry time.

‡ Adjusted for age, sex, body mass index, socioeconomic status, smoking status, alcohol status, Charlson comorbidity index, hemodialysis, antibiotic prescriptions, proton pump inhibitor use, corticosteroid use, other antibiotic allergies, nursing home resident, visits to general practitioner, and admissions to hospital.

### Mediation effects of alternative antibiotic use

Compared with patients who did not receive penicillins, patients receiving penicillins did not have an increased risk of MRSA (adjusted risk ratio 1.07, 95% confidence interval 0.95 to 1.20), but had an increased risk of *C difficile* (1.18, 1.06 to 1.31; supplemental table 4). Patients receiving macrolide antibiotics had an increased risk of MRSA (1.72, 1.54 to 1.91) and *C difficile* (1.30, 1.18 to 1.43). Patients receiving clindamycin had an increased risk of MRSA (2.97, 2.11 to 4.16) and *C difficile* (2.76, 2.00 to 3.81). Patients receiving fluoroquinolones had an increased risk of MRSA (2.38, 2.12 to 2.67) and *C difficile* (1.72, 1.54 to 1.93).

The effect of a penicillin allergy on the risk of MRSA was 55% mediated through β lactam alternative antibiotic classes; 55% mediated through fluoroquinolones, clindamycin, and macrolides; 54% mediated through fluoroquinolones and macrolides; 26% mediated through fluoroquinolones and clindamycin; and 24% mediated through fluoroquinolones alone (table 5). The effect of penicillin allergy on *C difficile* was 35% mediated through β lactam alternative antibiotic classes; 26% mediated through fluoroquinolones, clindamycin, and macrolides; 24% mediated through fluoroquinolones and macrolides; 20% mediated through fluoroquinolones and clindamycin; and 16% mediated through fluoroquinolones alone.

**Table 5:** Mediation analysis to estimate the indirect effect of listed penicillin allergy on meticillin resistant *Staphylococcus aureus* (MRSA) and *Clostridium difficile*

Mediating variables	MRSA				*C difficile*		
	Direct effect* risk ratio (95% CI)	Indirect effect† risk ratio (95% CI)	Percentage mediated		Direct effect* risk ratio (95% CI)	Indirect effect† risk ratio (95% CI)	Percentage mediated
All β lactam alternative antibiotics‡	1.41 (1.29 to 1.55)	1.36 (1.25 to 1.47)	55		1.34 (1.22 to 1.47)	1.12 (1.03 to 1.22)	35
Fluoroquinolones, clindamycin, and macrolides	1.41 (1.29 to 1.54)	1.36 (1.25 to 1.47)	55		1.36 (1.24 to 1.49)	1.09 (1.00 to 1.19)	26
Fluoroquinolones and macrolides	1.42 (1.30 to 1.56)	1.35 (1.24 to 1.47)	54		1.37 (1.25 to 1.50)	1.08 (1.00 to 1.18)	23
Fluoroquinolones and clindamycin	1.66 (1.50 to 1.83)	1.14 (1.04 to 1.25)	26		1.26 (1.13 to 1.39)	1.05 (0.96 to 1.15)	20
Fluoroquinolones	1.67 (1.52 to 1.85)	1.13 (1.03 to 1.23)	24		1.27 (1.14 to 1.41)	1.04 (0.95 to 1.14)	16

* The effect of penicillin allergy status on the risk of MRSA/*C difficile* not through a specific group of antibiotics adjusted for age, sex, body mass index, socioeconomic status, smoking status, alcohol status, Charlson comorbidity index, hemodialysis, antibiotic prescriptions, proton pump inhibitor use, corticosteroid use, other antibiotic allergies, nursing home resident, visits to general practitioner, and admissions to hospital.

† The effect of penicillin allergy status on the risk of MRSA/C difficile through a specific group of antibiotics adjusted for age, sex, body mass index, socioeconomic status, smoking status, alcohol status, Charlson comorbidity index, hemodialysis, antibiotic prescriptions, proton pump inhibitor use, corticosteroid use, other antibiotic allergies, nursing home resident, visits to general practitioner, and admissions to hospital.

‡ Includes fluoroquinolones, clindamycin, macrolides, aminoglycosides, vancomycin, and linezolid.

## Discussion

In this large cohort study reflective of the United Kingdom general population, we found that a penicillin allergy label was associated with a 69% increased risk of MRSA and a 26% increased risk of *C difficile*. Once documented, a penicillin allergy resulted in increased use of β lactam alternative antibiotics, with a fourfold increased incidence of macrolides and clindamycin utilization, and a twofold increased incidence of fluoroquinolone utilization. Furthermore, more than half of the increased MRSA risk and more than one third of the increased *C difficile* risk among patients with penicillin allergy was attributable to administered β lactam alternative antibiotics.

### Comparison with other studies and policy implications

We found that patients with a penicillin allergy label had nearly a 70% increased risk of new MRSA than their matched comparators, even after adjustment for known MRSA risk factors.^28^ This provides supporting evidence for a previous US study that showed a 14% increased MRSA prevalence in inpatients who were allergic to penicillin.^8^ Our result emphasises that outpatient use of antibiotics is strongly associated with the risk of developing MRSA.^22^ ^28^ Consistent with previous studies, we found that β lactam alternative antibiotics increased the risk of MRSA to a greater degree than did penicillins^12^ ^13^ ^29^ ^30^; whereas the mechanism of resistance is not known, the same factors that predispose staphylococcus to develop resistance to meticillin are thought to predispose staphylococcus to multidrug resistance that includes resistance to meticillin.^12^ ^29^ ^31^ With more than half of the increased MRSA risk among patients with listed penicillin allergy directly attributable to increased outpatient β lactam alternative antibiotic use (largely fluoroquinolones and macrolides), this risk appears modifiable if prescribing patterns among those with penicillin allergy could be altered.

*C difficile* is responsible for almost one half million infections and 15 000 deaths each year in the US, and the Centers for Disease Control and Prevention consider *C difficile* one of three urgent threats to public health.^32^ Patients with penicillin allergy in this study had a 26% increased *C difficile* risk compared with age, sex, and study entry time matched comparators after adjustment for other known risk factors for *C difficile*.^10^ ^33^ ^34^ ^35^ This result also corroborates the previous US study, which found a 23% increased *C difficile* prevalence in hospital patients with a penicillin allergy.^8^ While other studies similarly identified that clindamycin and fluoroquinolones were associated with the greatest risk of *C difficile*,^9^ ^10^ ^11^ we found that 35% of the heightened risk of *C difficile* in patients with penicillin allergy was directly attributable to use of β lactam alternative antibiotics, with quinolone use alone responsible for 16% of the heightened risk. The mechanism by which antibiotic use precipitates *C difficile* is through disruption of the host microbiome and creation of an environment where *C difficile* can overgrow.^36^ Antibiotics not captured in this dataset (eg, those administered at dialysis or in hospitals) and non-antibiotic risk factors^2^ ^34^ are likely responsible for the remainder of *C difficile* cases. Although current efforts to reduce *C difficile* largely focus on reducing *C difficile* infections in hospitals and rehabilitation centers, one third of *C difficile* infections occur in the community, and occur in outpatients.^37^ Our findings suggest that more systematic efforts to identify patients with listed penicillin allergy who are not truly allergic to penicillins could help decrease rates of community associated *C difficile*.

In this study, patients with documented penicillin allergy had an increased incidence of broad spectrum antibiotic use, including the extended Gram positive spectrum antibiotics vancomycin and linezolid, which should be reserved for patients with suspected or known MRSA (or vancomycin resistant enterococci for linezolid).^38^ ^39^ Use of the most narrow spectrum antibiotic that is effective for a given infection is a cornerstone of evidence based treatment for infection and is responsible antibiotic stewardship.^38^ Antibiotic stewardship committees enforce this aim in the hospital setting, with evaluations for penicillin allergy occasionally included in stewardship efforts.^40^ This analysis emphasises the importance of performing outpatient antibiotic stewardship and the role that penicillin allergy evaluations might play. Although diagnostic testing for penicillin allergy was developed in the 1960s, and has recently garnered the support of a variety of professional organizations,^38^ ^41^ ^42^ less than 0.1% of patients with a penicillin allergy label undergo confirmatory testing.^15^ Evaluation of penicillin allergy often involves a skin test, and if the result of skin testing is negative, a challenge dose of penicillin or amoxicillin is administered under medical observation.^15^ With these evaluation tools, evaluation of penicillin allergy has a more than 99% negative predictive value, takes less than three hours to perform, and costs about $220 (£165; €188; 2016 currency conversion).^15^ ^43^ Previous observational cohorts have shown that more than 90% of patients with listed penicillin allergies can be safely treated with penicillins.^14^ ^15^ ^40^

### Strengths and limitations of this study

In this study we used a representative population based cohort to increase the generalizability of our findings. Clinical data to characterise drug use, outcomes, and covariates were entered by physicians and captured electronically. The dataset used included granular allergy data linked to penicillin prescriptions and defined by type, severity, and certainty. Our study design used a comparator group who had recently been prescribed a penicillin but did not have a resultant penicillin allergy. Patients had high antibiotic use in the previous year since almost the entire cohort had recently had a penicillin (for infection) at baseline for cohort eligibility. Our GP practice based dataset could have missed the detection of some inpatient cases of MRSA and *C difficile;* however, these potential non-differential misclassifications would have biased our results towards the null, rendering our findings conservative. MRSA and *C difficile* were identified by physician diagnosis records. This approach has been successfully used in many previous epidemiologic studies,^13^ ^19^ ^20^ ^21^ ^22^ ^23^ ^24^ ^44^ ^45^ as microbial infections such as MRSA and *C difficile* are made objectively using highly accurate microbiologic and serologic tests. Although we used composite outcomes for MRSA and *C difficile* that were not restricted to infections, it is unlikely that GPs would screen asymptomatic patients and more likely that diagnoses occurred in relevant clinical contexts where infections were suspected. Further, our findings remained consistent and strong when we restricted the analyses to code subgroups suggestive of infections. Additionally, MRSA carriage alone is an important outcome that confers an increased risk of MRSA infection,^46^ and indicates antibiotic resistance—a healthcare priority throughout the world.^39^ ^47^ Finally, by choosing to study only the first documentation of MRSA and *C difficile*, we ensured capture of only new colonization or infection, which are clinically important outcomes. Although we controlled for many known potential confounders in these data, our observational study cannot rule out potential unknown or residual confounding.

### Conclusions

In this population based cohort study, a listed penicillin allergy was associated with a statistically significantly increased risk of MRSA and *C difficile* compared with patients matched by age, sex, and study entry time. Approximately one third to more than one half of this risk was attributed to use of non-β lactam antibiotics administered to outpatients. As infections with resistant organisms increase, systematic efforts to confirm or rule out the presence of true penicillin allergy may be an important public health strategy to reduce the incidence of MRSA and *C difficile*.

**What is already known on this topic**

Penicillin allergy is the most commonly documented drug allergy, reported by about 10% of patients
Although documented allergies impact prescribing behavior, a documented penicillin allergy does not often represent true, immediate hypersensitivity to penicillin
Previous studies have identified specific antibiotic uses that increase the risk of MRSA and *Clostridium difficile*

**What this study adds**

Patients with a documented penicillin allergy have an increased risk of new MRSA and *C difficile* that are modifiable, to some degree, through changes in antibiotic prescribing

## Web extra

Extra material supplied by authors

## Notes

Contributors: KGB, NL, YZ, RPW, and HKC designed the study. KGB and YL performed the literature review. KGB, NL, YZ, and HKC analyzed the data. KGB, NL, YZ, YL, RPW, and HKC analyzed and interpreted the results. KGB drafted the first report. NL, YZ, YL, RPW, and HKC assisted with interpretation and revision of the report. KGB and RPW obtained funding. KGB and HKC are guarantors of the study. All authors were involved in the review and approval of the manuscript. All authors are independent from funders. All authors had full access of the data in the study and can take responsibility for the integrity of the data and the accuracy of the data analysis. The corresponding author attests that all listed authors meet authorship criteria and that no others meeting the criteria have been omitted.

Ethical approval: This study was approved by THIN ethics review and deemed exempt by the Partners Human Research Committee.

Data sharing: No additional data available.

Transparency: The lead author (KGB) affirms that the manuscript is an honest, accurate, and transparent account of the study being reported. No important aspects of the study have been omitted. Any discrepancies from the study as planned have been explained.
