# Supplementary material for: Risk of meticillin resistant Staphylococcus aureus and Clostridium difficile in patients with a documented penicillin allergy: population based matched cohort study
Source: BMJ. 2018 Jun 27;361:k2400. doi: 10.1136/bmj.k2400 (PMC6019853; doi:10.1136/bmj.k2400)
Supplement: Supplementary file 1 — Supplemental information: tables 1-4 [file bluk042003.ww1.pdf]

Supplementary Appendix

Risk of methicillin-resistant *Staphylococcus aureus* and *Clostridium difficile* in patients with a documented penicillin allergy: A population-based matched cohort study

Kimberly G. Blumenthal, MD, MSc

Na Lu, MPH

Yuqing Zhang, DSc

Yu Li, MS

Rochelle P. Walensky, MD, MPH

Hyon K. Choi, MD, DrPH

Table of Contents

Supplemental Table 1. READ diagnosis codes used for primary exposure and outcomes..... 3

Supplemental Table 2. Impact of penicillin allergy on methicillin-resistant *Staphylococcus aureus* and *Clostridium difficile*, outcome detail 5

Supplemental Table 3. Impact of penicillin allergy on broad-spectrum beta-lactam alternative antibiotics infrequently administered to outpatients 7

Supplemental Table 4. Relationship between antibiotic use and the risk of methicillin-resistant *Staphylococcus aureus* and *Clostridium difficile* 8

Supplemental Table 1. READ diagnosis codes used for primary exposure and outcomes

Variable	Detail	READ Code
Penicillin allergy	Allergy	14L1.00, ZV14000
	Adverse reaction	TJ00.00, TJ00000, TJ00100, TJ00200, TJ00300, TJ00311, TJ00400, TJ00500, TJ00600, TJ00700, TJ00800, TJ00900, TJ00A00, TJ00B00, TJ00C00, TJ00D00, TJ00E00, TJ00F00, TJ00G00, TJ00z00, U600011, U600012, U600013, U600014, U600015, U600016, U600017, U600018, U600019, U60001A, U60001B, U60001C, U60001D, U60001E, U60001F, U60001G, U60001H, U60001I, U60001J
Methicillin-resistant *Staphylococcus aureus*	Methicillin resistant *Staphylococcus aureus* positive	4JP.00
	Methicillin resistant *Staphylococcus aureus*	A3B1100
	Multiple resistant *Staphylococcus aureus*	A3B1111

Supplemental Table 1. (cont.)

	Methicillin resistant *Staphylococcus aureus*	A3B1112
	MRSA infection of postoperative wound	SP25800
	MRSA-Multiple resistant staph aureus infection carrier	ZV02A00
	MRSA decontamination therapy	65c0.11
	Methicillin resistant *Staphylococcus aureus* eradication therapy	8BAk.00
Clostridium difficile	*Clostridium difficile* antigen detection	43k9.00
	Clostridium difficile toxin A detected	4JD2000
	Clostridium difficile toxin detection	683B.00
	Clostridium difficile infection	A3Ay200

Supplemental Table 2. Impact of penicillin allergy on methicillin-resistant *Staphylococcus aureus* and *Clostridium difficile*, outcome detail

**Outcome***	**Penicillin Allergy**		**Hazard Rate (Penicillin allergy compared to no allergy)**			
			Age, Sex, Entry-time-matched Unadjusted		Multivariable Adjusted†	
	**Yes**	**No**	HR (95%CI)	p-value	HR (95%CI)	p-value
MRSA Infection or Unspecified	420	865	1.86 (1.66 to 2.09)	<0.001	1.71 (1.52 to 1.92)	<0.001
MRSA Carrier	17	50	1.32 (0.76 to 2.28)	0.33	1.33 (0.76 to 2.31)	0.32
MRSA Eradication/Decontamination	6	11	2.08 (0.77 to 5.61)	0.15	1.98 (0.72 to 5.44)	0.19
**MRSA Total**	**443‡**	**926§**	**1.84 (1.64** to **2.06)**	**<0.001**	**1.69 (1.51** to **1.90)**	**<0.001**

C.difficile Infection	168	421	1.54 (1.29 to 1.84)	<0.001	1.39 (1.16 to 1.67)	<0.001
C.difficile Antigen/Toxin Detection[ǁ]	274	829	1.27 (1.11 to 1.46)	<0.001	1.18 (1.03 to 1.36)	0.02
***C.difficile* Total**	**442**	**1,250**[¶]	**1.37 (1.23** to **1.53)**	**<0.001**	**1.26 (1.12** to **1.40)**	**<0.001**

*MRSA Infection or Unspecified: 4JP.00, A3B1100, A3B1111, A3B1112, SP25800; MRSA Carrier: ZV02A00; MRSA Eradiation/Decontamination: 65c0.11, 8BAk.00; *C.difficile* Infection: A3Ay200; *C.difficile* Antigen/Toxin Detection: 43k9.00, 4JD2000, 683B.00.
[†]Age-, sex-, entry time-matched and adjusted for age, sex, body mass index, socioeconomic status, smoking alcohol, Charlson comorbidity index, hemodialysis, antibiotic prescriptions, proton pump inhibitor use, corticosteroid use, other antibiotic allergies, nursing home living, general practitioner visits, and hospitalizations.
[‡] One patient had more than one diagnosis the same day
[§]Three patients had more than one diagnosis the same day
[¶] Four patient had more than one diagnosis the same day
Abbreviations: HR, hazard rate; MRSA, methicillin-resistant *Staphylococcus aureus*; *C.difficile, Clostridium difficile*

Supplemental Table 3. Impact of penicillin allergy on broad-spectrum beta-lactam alternative antibiotics infrequently administered to outpatients

	**Antibiotic Use Frequency (Events/ 1,000 person-years)**		**Incidence Rate Ratio (Penicillin allergy compared to no allergy)**			
	Penicillin Allergy	No Penicillin Allergy	Age, Sex, Entry-time-matched		Multivariable Adjusted*	
	(n=64,141)	(n=237,258)	IRR (95%CI)	p-value	IRR (95%CI)	p-value
Vancomycin	0.27	0.11	2.51 (1.96 to 3.22)	<0.001	2.24 (1.75 to 2.88)	<0.001
Aminoglycosides	0.66	0.56	1.16 (1.01 to 1.33)	0.04	1.09 (0.94 to 1.25)	0.26
Linezolid	0.06	0.01	5.36 (2.78 to 10.35)	<0.001	5.07 (2.61 to 9.82)	<0.001

*Adjusted for age, sex, body mass index, socioeconomic status, smoking alcohol, Charlson Comorbidity Index, hemodialysis, antibiotic prescriptions, proton pump inhibitor use, corticosteroid use, other antibiotic allergies, nursing home living, general practitioner visits, and hospitalizations.
Abbreviations: IRR, incidence rate ratio; CI, confidence interval

Supplemental Table 4. Relationship between antibiotic use and the risk of methicillin-resistant *Staphylococcus aureus* and *Clostridium difficile*

**Antibiotics**			**MRSA**		***C.difficile***	
		N	Cases	RR (95% CI)*	Cases	RR (95% CI)*
Beta-Lactams						
Penicillins	No	101,444	409	1.07 (0.95 to 1.20)	475	1.18 (1.06 to 1.31)
	Yes	199,955	956		1,213	
Cephalosporins, 1st Generation	No	247,774	926	1.78 (1.59 to 2.01)	1,183	1.64 (1.47 to 1.82)
	Yes	53,625	439		505	
Beta-Lactam Alternatives						
Macrolides	No	186,863	646	1.72 (1.54 to 1.91)	912	1.30 (1.18 to 1.43)
	Yes	114,536	719		776	
Clindamycin	No	299,403	1329	2.97 (2.11 to 4.16)	1,649	2.76 (2.00 to 3.81)
	Yes	1,996	36		39	
Fluoroquinolones	No	257,925	898	2.38 (2.12 to 2.67)	1,237	1.72 (1.54 to 1.93)
	Yes	43,474	467		451	

*Adjusted for age, sex, body mass index, socioeconomic status, smoking alcohol, Charlson Comorbidity Index, hemodialysis antibiotic prescriptions, proton pump inhibitor use, corticosteroid use, other antibiotic allergies, nursing home living, general practitioner visits, and hospitalizations.

Abbreviations: MRSA, methicillin-resistant *Staphylococcus aureus*; *C.difficile*, *Clostridium difficile*; RR, risk ratio; CI, confidence interval
